# A case report on Madelung’s disease and comprehensive review of the literature

**DOI:** 10.1186/s13023-024-03303-w

**Published:** 2024-08-17

**Authors:** Cheng Jiao, Wei Liu, Yiming Qiao, Shuai Qi, Yifei Shen

**Affiliations:** Department of General surgery, Bethune International Peace Hospital, No. 398, Zhongshan West Road, Qiaoxi District, Shijiazhuang, 050051 Hebei Province China

**Keywords:** Benign symmetric lipomatosis, Lipid metabolism, Liposuction surgery, Madelung’s disease, Surgical treatment

## Abstract

**Background:**

Madelung’s disease (MD), alternatively referred to as benign symmetric lipomatosis, multiple symmetric lipomatosis, or Launois–Bensaude syndrome, is an uncommon benign disorder marked by symmetric proliferation of adipose tissue in the head, neck, and torso regions.

**Case description:**

In this case, the patient was a female with the late middle-aged demographic, diagnosed with Type I MD. Notably, she exhibited no prior history of alcohol consumption, and there was no family history of MD. Subsequent to the clinical diagnosis, the patient underwent medical imaging assessments to delineate the surgical parameters. Post-surgery, she demonstrated a favorable recovery trajectory, marked by the absence of any surgical complications.

**Result:**

The patient underwent successful surgical excision of the lipomatous mass. Postoperatively, she had an uneventful recovery with no complications and no recurrence observed during the follow-up period of seven months.

**Conclusion:**

Timely diagnosis and early surgical intervention play a pivotal role in enhancing the quality of life for individuals with MD. Preoperative medical imaging examinations function as highly effective tools, contributing to heightened surgical safety and a decreased probability of encountering complications during the surgical procedure.

## Introduction

Madelung’s disease (MD), also recognized as benign symmetric lipomatosis, multiple symmetric lipomatosis, or Launois–Bensaude syndrome, is an uncommon benign condition marked by the progressive and symmetrical accumulation of anomalous adipose tissue in various regions, including the neck, shoulders, upper arms, chest, back, and other trunk areas [1]. In severe instances, MD can result in impairment of trunk movement, dyspnea, and dysphagia. The condition predominantly affects males, with few occurrences reported in females, and is most frequently observed in individuals of Mediterranean descent. However, the etiology and pathogenesis of MD remain unclear. A noteworthy association has been observed between MD and alcohol consumption, with alcohol abuse reported in a majority of patients [2, 3]. 

MD was initially documented by Brodie [4] in 1864, and later analyzed and consolidated by Madelung [5] in 1888, who subsequently named the condition Madelung’s disease. To date, approximately 600 cases have been reported in various countries, including China. MD primarily manifests in regions such as the occiput, shoulders, chest, and submandibular area, with the head and neck being the most frequently affected areas. Key features of MD include the presence of lipomas, symmetrical distribution, localized diffusion, and a tendency to occur on the face and neck.

In most of the cases of MD, patients typically display mild symptoms characterized by physical deformities and limited neck movement, often with a gradual progression of the condition. However, in severe instances, the lipomatous mass can exert pressure on the trachea and esophagus, resulting in compromised respiratory and swallowing functions. MD has been associated with comorbidities such as diabetes mellitus, hyperlipidemia, hyperuricemia, and neurological disorders.

A retrospective analysis by Li et al. [6]. involving 54 patients with MD revealed that endocrine system diseases were the most prevalent comorbidities, accounting for 81.48% of cases. Notably, 20.37% of these patients were diagnosed with cancer, particularly tumors related to the digestive system. Moreover, as reported [7], the incidence of liver diseases, particularly chronic alcoholic liver disease, is notably high among patients with MD.

The primary treatment modality for this condition is predominantly surgical excision. However, the challenge lies in achieving complete excision of lipomas due to their inclination to enwrap or infiltrate critical organs and anatomical structures in the head and neck. The surgical procedure is complex, often accompanied by a high incidence of complications. Additionally, the disease exhibits a notable propensity for postoperative recurrence.

Given the low incidence rate of the disease, there is a scarcity of related reports both in China and other countries. In September 2023, our department encountered a case of MD. This paper aims to present the diagnostic and therapeutic details of this specific case.

## Method

### Patient data

A 55-year-old female patient presented with a neck mass that had been observed for over 3 years. Initially painless and without associated movement impairment, dysphagia, or other discomfort, the patient did not seek medical evaluation or treatment at the onset. As the mass progressively increased in size and became painful, the patient sought surgical treatment at our department. Before the onset of this condition, the patient had no history of hypertension, coronary heart disease, diabetes mellitus, or other underlying conditions. There was no specific medication usage or alcohol consumption, and her family members had no similar illness histories.

### Medical imaging and diagnosis

Upon specialist examination, a subcutaneous mass was observed in the posterior aspect of the patient’s neck, symmetrically distributed on both sides, measuring approximately 30 cm × 20 cm. The mass displayed a soft texture with well-defined boundaries and exhibited good mobility upon palpation. The patient reported slight pain upon palpation of the mass, and there were no signs of redness or ulceration in the surrounding skin.

**Fig. 1:**
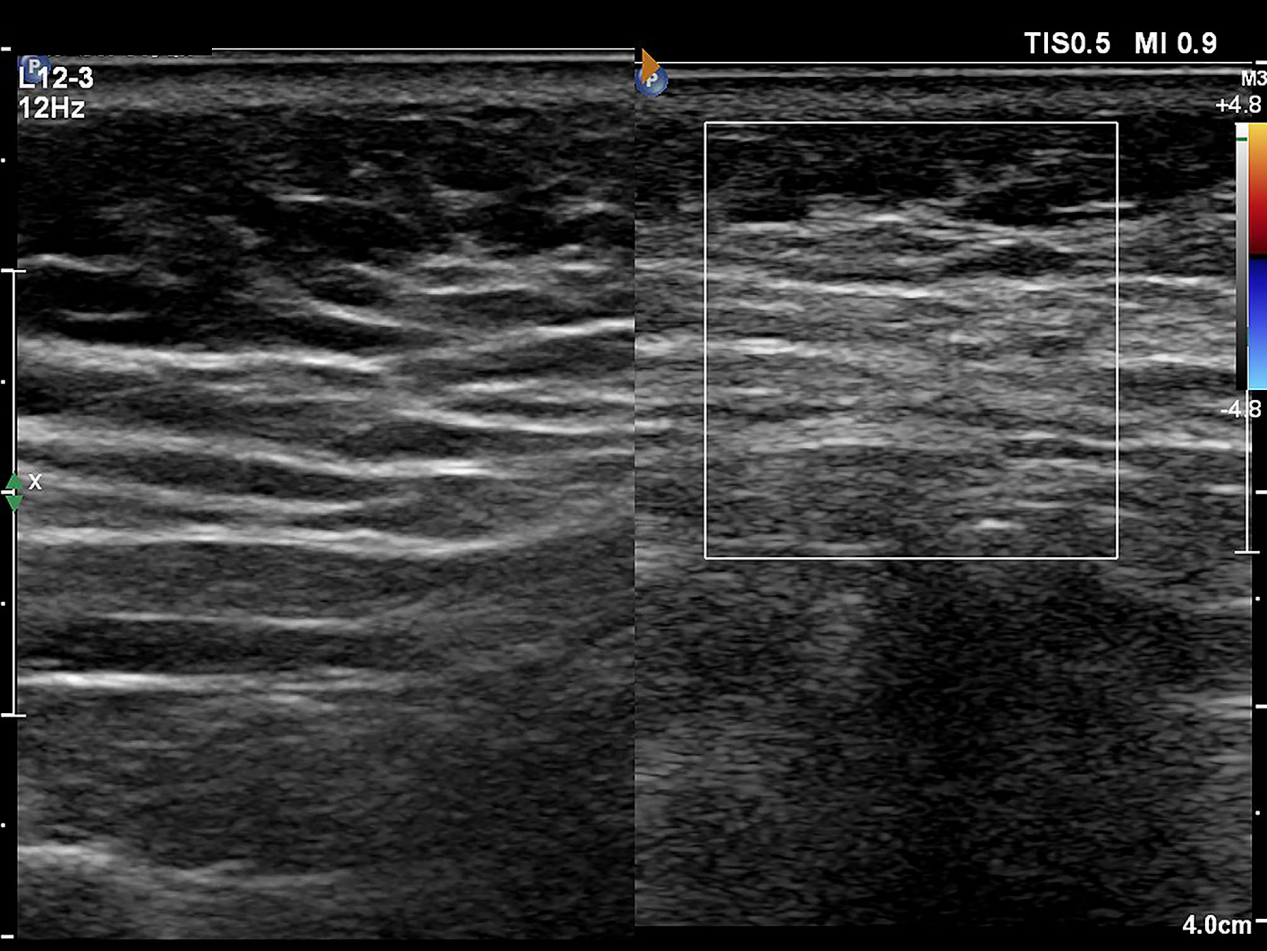
Ultrasonography imaging of the neck mass

**Fig. 2:**
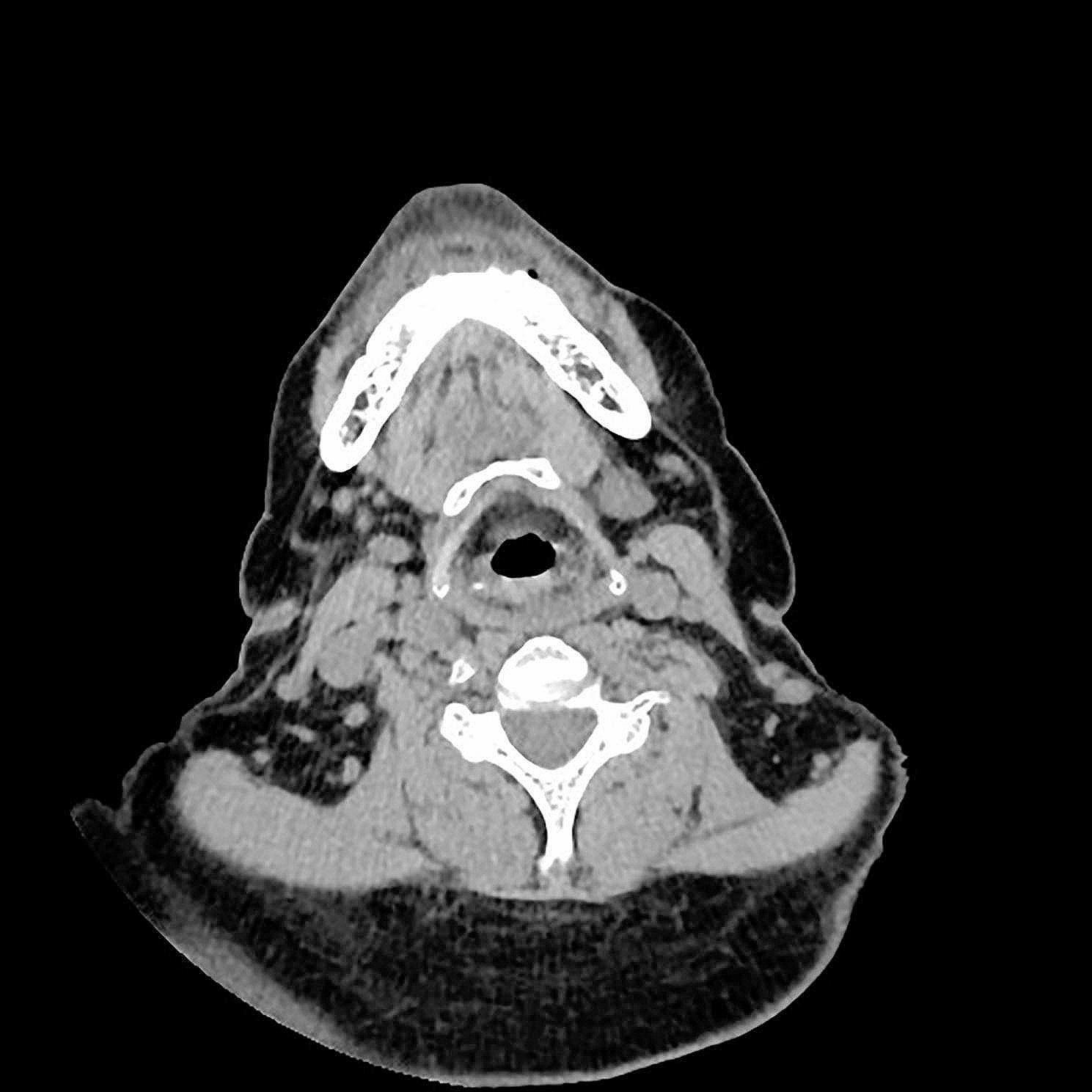
Neck mass evaluation through CT imaging

Medical imaging examinations, including color Doppler ultrasound and computed tomography (CT) of the neck and chest, revealed diffuse abnormal thickening in the subcutaneous fat layer of the neck, consistent with findings suggestive of MD. Based on the comprehensive evaluation of the medical history, physical examination, and medical imaging results, the patient received a diagnosis of MD(Figs. 1 and 2).

**Fig. 3:**
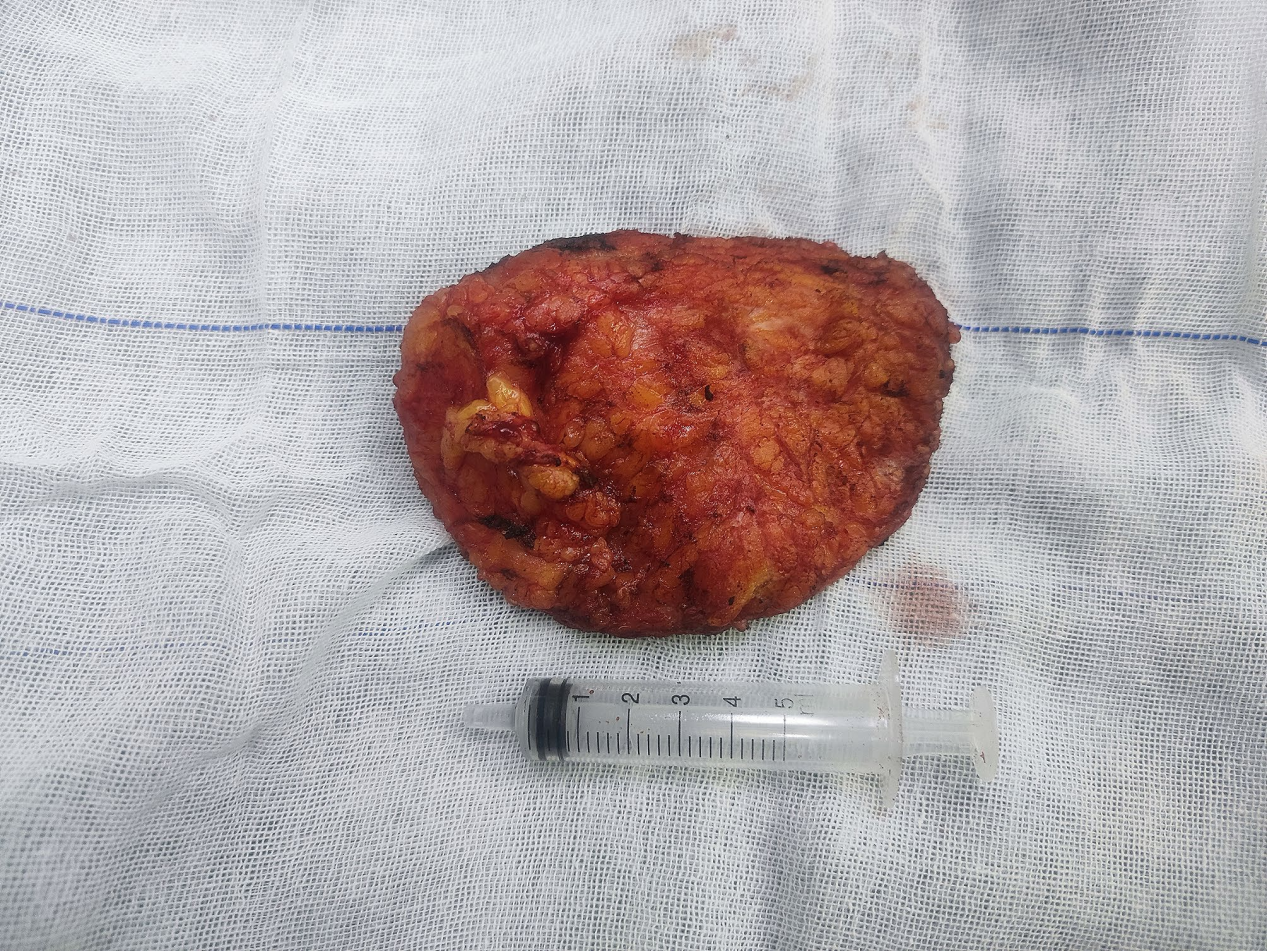
Specimen excised from the lesion area

**Fig. 4:**
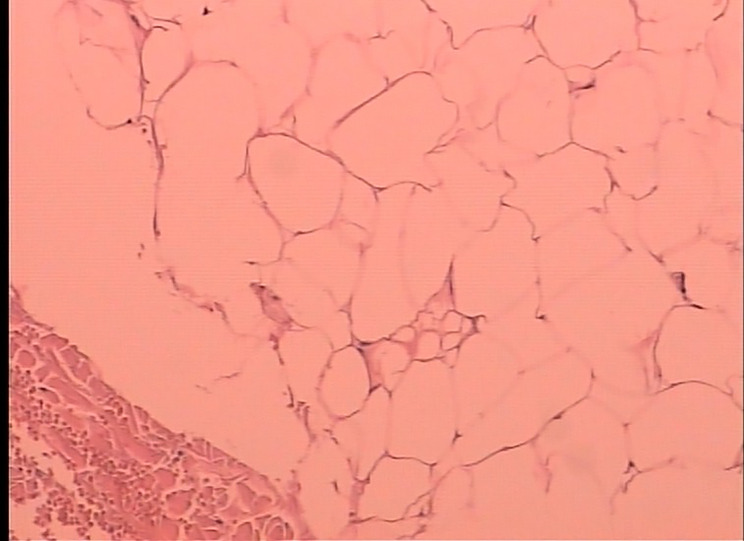
Pathological examination of the patient revealed a substantial presence of adipocytes, which are stained with paraffin and observed at a magnification of ×400

### Surgical procedure

The patient underwent thorough examinations to eliminate surgical contraindications and subsequently had the mass excised from the posterior aspect of the neck under local anesthesia. The surgical procedure proceeded without complications. Intraoperatively, a diffuse lipoma-like mass (Fig. 3) was observed exhibiting unbounded growth within the subcutaneous fat layer. Following excision, a negative pressure drainage tube was placed at the operative site along with absorbent dressings, and the wound was securely wrapped.

### Postoperative care

Postoperative care entailed meticulous dressing changes, periodic replacement of the negative pressure drainage device, continuous monitoring of drainage fluid characteristics, and removal of the drainage tube after one week. Suture removal occurred gradually to prevent premature wound dehiscence.

## Result

Subsequent to surgery, the patient experienced a successful recovery. The pathology report identified the excised mass as adipose tissue proliferation (Fig. 4). The patient was discharged without complications 10 days post-surgery and displayed no abnormalities during the three-month and seven-month follow-up. The postoperative period was marked by the absence of complications, and the patient reported significant improvement in symptoms, with no recurrence of the lipomatous mass observed during the follow-up period.

## Discussion

Sporadic cases of Madelung’s disease (MD) tend to affect middle-aged men more frequently, with a male-to-female ratio of 14.6:1, and are associated with a history of alcohol abuse [8]. However, in this particular case, the patient is a female with no pre-existing underlying conditions or history of alcohol abuse, making this clinical presentation relatively uncommon.

The etiology and pathogenesis of MD remain elusive. Some scholars postulate that alcohol may impact mitochondria and chromosomes, leading to dysfunction and subsequent abnormalities in lipid metabolism within adipose tissue, ultimately promoting lipoma growth [9]. Other studies suggest that the association between alcohol consumption and MD may involve defective brown adipocyte function [10], as well as impaired breakdown and metabolism of brown adipose tissue under the influence of catecholamines. Notably, despite men being more susceptible to MD, mitochondrial DNA mutations and nuclear genes involved in mitochondrial maintenance do not exhibit a male bias in familial cases. In clinical settings, MD is typically categorized into three main types. Type I predominantly affects males and manifests as symmetrical, round, non-encapsulated fat masses distributed on the body surface, with a notable concentration in the upper body. The distal parts of the limbs remain unaffected. Type I MD may be accompanied by associated complications. Notably, fat accumulation in the mediastinum has the potential to compress the superior vena cava and the recurrent laryngeal nerve in the trachea and esophagus, resulting in corresponding symptoms [11]. Moreover, infiltration of adipose tissue into the tongue may give rise to macroglossia, characterized by the enlargement of the tongue extending into the floor of the mouth, subsequently causing restricted tongue movement and dysphagia. Deposition of lipomatous tissues in the pharynx can potentially lead to obstructive sleep apnea-hypopnea syndrome [12]. Although the invasion of lipomatous tissues into the parotid glands and salivary glands is relatively uncommon, it may coincide with dyspnea [13]. 

Type II MD does not exhibit a gender preference, displaying a comparable incidence rate among males and females. It primarily affects the upper back, deltoid region, upper arms, buttocks, and upper thighs, among other areas, and may also involve fat accumulation in the upper abdomen in some patients.

Type III MD is congenital and primarily involves the trunk, often occurring in children. Some scholars propose a four-type classification of MD, which includes a distinct type differentiating lipomatosis impacting visceral organs from the aforementioned three types [14]. 

The diagnosis of MD primarily relies on the patients’ clinical presentation along with imaging examinations. Color Doppler ultrasonography provides high-resolution imaging of superficial soft tissue, enabling clear visualization of lesion blood flow and surrounding vascular distribution. Ultrasonic manifestations of MD typically involve abnormal accumulation and thickening of subcutaneous and intermuscular adipose tissues in the neck regions, without distinct or specific echo signals.

Computed tomography (CT) emerges as the preferred imaging modality due to its capacity to offer precise details regarding lesion location, size, nature, and anatomical relationship with surrounding tissues. Such information is crucial for surgical planning and defining the operative scope [15]. In this case, diffuse lipomatous lesions lacking defined borders were identifiable via ultrasound. The utilization of preoperative ultrasound localization and marking played a pivotal role in assessing the extent of excision, planning the surgical incision, and avoiding vascular structures to minimize intraoperative bleeding.

Surgery is presently the primary treatment modality for managing MD, encompassing options such as excision surgery and liposuction surgery. Excision surgery is the preferred method for addressing MD, although its efficacy is limited by the absence of well-defined capsules within MD lesions, unclear boundaries, extensive excision requirements, and a notable recurrence rate. Due to the rarity of Madelung’s disease and the diverse surgical conditions among patients, there is a dearth of reports on the recurrence time following surgery. As a result, no comprehensive analysis exists on this topic. Individual case reports typically opt for a follow-up period of 3–6 months, which further underscores the need for more extensive research in this area. Consequently, surgical excision primarily aims at symptom improvement rather than achieving complete lesion removal. Liposuction surgery, characterized by a relatively straightforward procedure, is more suitable for cases involving extensive lesions or patients in suboptimal health conditions who may not tolerate excision surgery well. Systematic evaluations have shown that, in comparison to excision surgery, liposuction surgery results in fewer complications. However, it is important to note that the recurrence rate tends to be higher after liposuction surgery [16]. 

Common complications associated with excision surgery include local hematoma, subcutaneous fluid accumulation, and delayed wound healing. Studies have indicated the significant role of intraoperative tumescent solution use [17], a combination typically comprising saline solution, anesthetic drugs, and hemostatic agents. This solution aids in gradually separating fat cells and facilitates smoother surgery, thereby reducing these surgical complications.

In instances where surgical intervention is not feasible, drug therapy emerges as a viable alternative. Therapeutic drugs commonly employed include salbutamol, promoting the breakdown of adipose tissue, and a combination of vitamins C and E, coenzyme Q10, and levocarnitine, aiming to restore mitochondrial function. MD is characterized by a high recurrence rate, with age, body mass index, and alcohol intake identified as key risk factors for recurrence. It is imperative for individuals with MD to control their alcohol consumption as a crucial measure in preventing recurrence [18]. 

## Conclusion

The patient in this case is a female diagnosed with Type I MD with no indication of alcohol dependence; a clinical rarity rarely documented in existing literature. Post-surgical excision, the intervention exhibited a favorable outcome devoid of complications, highlighting the pivotal role of prompt diagnosis and timely surgical intervention in maintaining a superior quality of life for patients with MD. The incorporation of preoperative imaging assessments holds the potential to significantly enhance surgical safety and reduce the likelihood of complications during the procedure. As research into the pathogenesis of MD progresses, the prospect of more efficacious treatment modalities emerging in the future has become increasingly plausible.

## Data Availability

The datasets of the study are not publicly available due to patient privacy and confidentiality. Anonymized data can be made available from the corresponding author upon reasonable request.

## Abbreviations

MD: Madelung’s disease
BMI: Body Mass Index
